# Insulin smart drug delivery nanoparticles of aminophenylboronic acid–POSS molecule at neutral pH

**DOI:** 10.1038/s41598-021-01216-3

**Published:** 2021-11-08

**Authors:** Won Jung Kim, Yong-Jin Kwon, Chung-Hyun Cho, Sang-Kyu Ye, Kyu Oh Kim

**Affiliations:** 1 Department of Fiber-System Engineering, Dankook University, 152, Jookjeon-ro, Suji-gu, Yongin-si, Gyeonggi-do 448-701 Republic of Korea; 2 Departments of Pharmacology, Seoul National University College of Medicine, Seoul, South Korea

**Keywords:** Nanoparticles, Drug delivery, Biomedical materials

## Abstract

Self-regulated “smart” insulin administration system that mimic pancreatic endocrine function would be highly desirable for diabetes management. Here, a glucose-responsive continuous insulin delivery system is developed, where novel polyhedral oligosilsesquioxane (POSS) modified with 3‐aminophenylboronic acid (APBA) were used to encapsulate insulin (insulin entrapment efficiency: 73.2%) to prepare a fast response, high stability, good distribution, and excellent biocompatible system. Due to the strong hydrophobicity of POSS, the POSS moiety is located at the core in aqueous solution and combines with the boronic group of APBA and the diol generated in PEG-insulin to form a nanomicelle structure, that is, nanoparticles naturally. Micelles self‐assembled from these molecules possess glucose‐responsiveness at varying glucose concentrations. The interaction of the PBA and diol containing insulin via boronate ester bond and its interchange with glucose was investigated by FT-IR, ^1^H NMR and XPS. Furthermore, the successful glucose-triggered release of insulin from the POSS-APBA micelles was investigated at neutral pH. A linear graph was plotted with the measured released insulin vs glucose concentrations, with a linear correlation coefficient (R2) value close to 1. Circular dichroism (CD) spectroscopy analysis was performed to measure insulin activity by comparing secondary structures of insulin, PEG-Insulin, and POSS-APBA@insulin. When confirming intracellular apoptosis signaling, cleaved caspase 3 and caspase 9 were not increased by 640 μg/ml POSS-APBA and POSS-APBA@insulin in HeLa, HDF and HUVE cells. Application in the biomedical field for controlled delivery of insulin appear to be promising.

## Introduction

Diabetes which is a metabolic disease characterized by hyperglycemia, and the high blood sugar causes various symptoms and complications to detriment the quality of life. A complete cure still needs to be developed. In the case of type 1 diabetes, the best known therapeutic method is for the patient to check the blood sugar level with a biosensor and inject insulin or other hypoglycemic agent, although it is not a complete remedy. However, administration of the drug by injection may drastically lower the blood sugar level and may even bring about life-threatening situations. The patient has to take painful injections several times a day, even if they do not want to get injections. Various insulin administration methods are being studied worldwide to overcome this problem. Typical methods are pulmonary delivery^1,2^, transdermal delivery using microneedles^3,4^ and oral delivery^5^. However, insulin may be denatured or loss during administration may result in lower application to the human body when these methods are used. A recent development is the self-regulated glucose-responsive insulin delivery system by which a single administration lasts for a long time and autonomously delivers the drug in response to the change in the blood sugar level to maintain a normal blood sugar level^6–9^.

Typical glucose-responsive moieties are glucose oxidase (GOx)^10,11^, phenylboronic acid (PBA)^12,13^ and glucose binding protein (GBP)^14,15^. Enzymes and proteins oxidize in air resulting in loss and handling problems. PBA and its derivatives known to be capable of a reversible boronate group formation exhibit a high affinity towards cis-diol compounds and it is well known that a PBA functionalized moiety may be utilized as a glucose sensitive drug delivery system^16^. However, a problem exists in that PBA exhibits the affinity only in an alkaline conditions of pH greater than 8.5. In order to obtain a stable affinity even in neutral pH, amino groups may be introduced to boric acid groups to protect the boric acid-polyol bond from the nucleophilic attack by water molecules^17^. However, the amino group in the boric acid containing moiety is highly reactive and may decrease the yield in the synthesis of other functional material. Our new approach is to assemble boronic acid with POSS inorganic molecules having nanopores in a physiologic condition. POSS endows the assembly with the essential properties of a micelle due to the following reasons. (1) The structure of POSS is a three dimensional sphere and contains 8 alkyl groups allowing it to be soluble in organic solvents facilitating the reaction/assembly with organic compounds. (2) The hydrophobic effect of the phenyl groups of the phenylboronic acid is minimized by the steric hindrance of POSS. As a result a large space is formed where the three dimensional glucose and insulin may easily undergo interchange reactions allowing high yield reactions even with small amounts. (3) Stable boric acid-polyol reaction are made possible under neutral pH conditions (4) The denaturalization of the protein drug is minimized by enhancement of the physicochemical stability through the loading of the protein insulin drug onto POSS. (5) The loading efficiency of the hydrophobic drug is high^18–20^.

The self‐assembly of poly (ethylene glycol) (PEG) and polyhedral oligosilsesquioxane (POSS) nanoparticles demonstrated in our earlier work was used to encapsulate insulin for oral drug delivery carriers which can respond to pH^19^. Prior studies have been conducted on the derivative of insulin using the amino group of insulin^21,22^. We used PEG-insulin induction, in which PEG was bound to insulin^23^. PEG has features such as high compatibility and stability. A PEG-Insulin derivative was prepared by linking the amino group of insulin with the hydroxyl group of PEG^24,25^. We prepared POSS-APBA@insulin conjugated with POSS-APBA using the diol of PEG bound to the alpha and beta chains adjacent to Insulin. In this study, we report on the design of a POSS based organic–inorganic hybrid smart drug delivery system which reacts sensitively to glucose in in-vivo pH conditions and releases insulin through an interchange reaction. The structure and reactions of the synthesized drug delivery system was characterized by ^1^H NMR, FT-IR, XPS and TGA, and the particle shape and size were studied using SEM and TEM. Zeta potential measurements were made to study the effect of pH on the potential, size, PDI dispersity, particle mobility and conductivity. Circular dichroism (CD) spectroscopy analysis was performed to evaluate the conformational stability of insulin. The biocompatibility was also studied to explore the applicability as an innocuous hybrid nano drug delivery system.

## Materials and methods

### Materials

PSS-[2-(3,4-epoxycyclohexyl)ethyl]-heptaisobutyl substituted (POSS, Empirical Formula: C_36_H_76_O_13_Si_8_, MW:941.66), 3-Aminophenylboronic acid monohydrate (APBA, purity:98%), dimethyl sulfoxide (DMSO), insulin human (Empirical Formula:C_257_H_383_N_65_O_77_S_6_, MW:5807.57), hydrazine monohydrate, ammonia solution, polyethylene glycol (Mw = 200, PEG), tetrahydrofuran (THF), and sodium cyanoborohydride were purchased from Sigma Aldrich (Seoul, Korea) and used as received.

### Synthesis of POSS-APBA

Synthesis of POSS-APBA is carried out in three steps. First 10.0 mg POSS and 1.67 mg APBA is put in 9 ml THF and stirred at 50 °C for 3 h in an oil bath to make a homogeneous solution. To this solution 50 μl of hydrazine monohydrate catalyst and 50 μl of ammonia solution are slowly added and the mixture allowed to react for 1 h., then cooled to room temperature. The POSS-APBA product solution was placed in a vacuum oven at 45 °C to remove the THF solvent and obtain powders. The POSS-APBA powder was washed in aceton to remove the unreacted reactants, filtered using a vacuum filter on the membrane filter and stored in a dessicator at room temperature.

### Synthesis of PEG-insulin

To introduce diol groups to insulin, 8.0 mg insulin and 1.0 g PEG were dissolved in 10 ml DMSO to which 0.1–0.2% sodium cyanoborohydride catalyst was added and stirred for 1 h. then stabilized at room temperature for 1 h.

### Preparation of POSS-APBA@insulin

The preparation of POSS-APBA@insulin is carried out in two steps, synthesis and solvent exchange. In the synthesis step, 11 mg of prepared POSS-APBA powder and the PEG-insulin DMSO solution are stirred for 2 h and then stabilized for 0.5 h. The stabilized solution in DMSO is changed to an aqueous solution in the solvent exchange step by dialysis. The criteria for the selection conditions of dialysis tubes used in dialysis selected the size that the POSS-APBA@insulin nanoparticles we synthesized could not pass through the tube and only the solvent could pass through it. The stabilized solution is put in a dialysis tube with an MWCO size of 100-500D, and the tube is immersed in a water bath filled with distilled water for dialysis to remove organic solvent. In order to minimize the denaturation of insulin from effect of temperature and light, we have performed dialysis in a refrigerator at 3℃ and with blocking out light use aluminum foil. And then the mixture was poured into dialysis tube (spectra/Por 6, MWCO: 6.0 kD) and dialyzed against distilled water under magnetic stirring for 1 day in order to remove all residues. The distilled water in the bath is replaced with fresh distilled water at 0.15, 0.3, 0.45, 1, 3, 6, 12, and 24 h. to increase the dialysis efficiency. After dialysis, the POSS-APBA@insulin solution is stored in a refrigerator at 3℃.

## Characterization

### Fourier-transform infrared spectroscopy (FT-IR)

Fourier transforms infrared (FT-IR) spectra analysis was obtained on a Perkin Elmer Spectrum II, for the confirmation of synthesis and the structural analysis of the sample. The FTIR spectra were obtained from KBr pellets. The KBr pellets were made by mixing KBr powder and the sample at a ratio of about 100:1, using agate induction so that it is well dispersed, and then making a pellet by applying pressure on a pellet molding apparatus. The sample was analyzed in the range of 4000 –400 cm^−1^ to confirm the presence of characteristic peaks of POSS-APBA and the changes in peak positions and shapes.

### ^1^H-Nuclear magnetic resonance spectrometer (^1^H-NMR)

^1^H-NMR analysis was performed for structural analysis of the synthesized material. To completely remove the solvent from the POSS-APBA, POSS-APBA@insulin, and POSS-APBA@glucose samples, samples were dried in a vacuum oven at 90 °C for 24 h. The samples were dissolved in deuterated chloroform at a concentration of 0.02 g/0.7 ml and analyzed.

### X-ray photoelectron spectroscopy (XPS)

XPS analyses was carried out with microfocus monochromatic x-ray source: Al-K_α_ (1486.6 eV), energy resolution (Ag 3d5/2): ≤ 0.5 eV, sensitivity : 4,000,000 cps, ultimate vacuum : < 5.0 × 10^–9^ mbar, X-ray spot size: 10 μm ~ 400 μm, analyzer type: double-focusing, hemispherical analyzer with 128-channel detector, depth profiling: MAGCIS dual mode ion source.

### Thermogravimetric analysis (TGA)

Thermal stability analysis of POSS, APBA and synthesized POSS-APBA was carried out using TGA N-1000. The weight of the samples were 6.41 mg for POSS, 5.55 mg for APBA, and 7.41 mg for POSS-APBA. The samples were heated from 20 to 700 °C at a rate of 10 °C/min.

### Scanning electron microscope (SEM) & transmission electron microscope (TEM)

The three-dimensional structure and shape of the particles were confirmed with SEM, S-5200 and TEM, JEM-1011. The samples were prepared by dropping POSS-APBA and POSS-APBA@insulin, POSS-APBA@glucose nano particles stored in distilled water on carbon tape, drying the tape in a vacuum oven at 80 °C for 24 h, and then coating the carbon tape for SEM observation. Transmission electron microscope (TEM) images were obtained using a JEM-1011, Philips CM200 transmission electron microscope operating at 40 to 100 kV.

### Zeta potential (ζ)

The zeta potential was measured to determine the variation in the stability of POSS-APBA and POSS-APBA@insulin at different pH buffer and 37 °C. The zeta potential measurement was carried out on a ELSZ-2000S (Otsuka, Japan) equipment with the samples dispersed in distilled water where the pH was controlled with 0.1 N HCl and 0.1 N NaOH. Zeta-potential is difficult to measure directly, and the usual route is to acquire this data indirectly from the measurement of the electrophoretic mobility (particle velocity divided by the electric field strength) under an applied electrical field according to Henry's equation (Eq. 1):

1 $$\frac{U}{E} = \frac{2\varepsilon \upzeta \text{F(ka)}}{{3\upeta }}$$

where, U/E is the electrophoretic mobility (m^2^ s^−1^ V^−1^), ζ is the zeta-potential (V), ε is the solvent dielectric permittivity (or constant) (kg m V^−2^ s^−2^), η is the viscosity (kg m^−1^ s^−1^), particle size to the Debye length, 1/κ. Thus, κa ≫ 1 indicates that the particle radius (a) is large compared to 1/κ (1/κ is ∼10 nm for 1 mM aqueous salt solutions)^26^.

### Zeta sizer

Zeta sizer Nano ZS90 (Malvern Instruments, Worcestershire, UK) was used to measure the particle size and behavior of POSS-APBA and POSS-APBA@insulin under various pH conditions and sugar concentrations at 37 °C. Zeta-size was measured with Zeta sizer Nano ZS90 (Malvern instruments, Worcestershire, UK), and each sample was dispersed in distilled water. When measuring the Zeta-size, hydrochloric acid and sodium hydroxide were added to the distilled water solution to adjust the pH to 2, 4, 6, 7, 8, and 10. The measurements were carried out at low, middle and high glucose concentrations.

### Circular dichroism spectroscopy (CD)

The secondary structure of insulin in PEG-insulin and POSS-APBA@insulin particles were evaluated to determine the effect of PEG and POSS-APBA particles on the secondary structure of insulin. CD spectra was obtained on a circular dichroism spectrometer, Jasco J-150. Analysis was carried out on three samples in the wavelength range of 190–260 nm with the scanning speed of 100 nm/min at 25 °C with 200 μl samples in a 1 mm path cell and the average values are reported. Three samples, human insulin, PEG-insulin, and POSS-APBA@insulin, were analyzed using phosphate buffer saline solution (PBS pH 7.4) as solvent. The concentration of human insulin and PEG-insulin was adjusted to 0.5 mg/ml by diluting a stock solution whose concentration was 1 mg/ml with PBS, and in the case of POSS-APBA@insulin the concentration was adjusted to 0.3 mg/ml by diluting a stock solution whose concentration was 0.7 mg/ml with PBS. The spectra obtained were compared with each other. Additionally, the final mdeg value obtained was compensated using the molar ellipticity value before carrying out the analysis. The equation used for compensation is below:

2 $$\left[ \theta \right]\left( {\deg \cdot {{cm}}^{2} \cdot {{dmol}}^{ - } } \right) = \frac{{{{Ellipticity}}\left( {m\,deg } \right) \cdot 10^{6} }}{{{{pathlength}}\left( {{{mm}}} \right) \cdot \left[ {{{Protein}}} \right]\left( {\mu M} \right) \cdot \left( {n - 1} \right)}}$$

The data was also analyzed after applying a deconvolution program.

### In vitro drug loading efficiency

To determine the loading efficiency of insulin in POSS-APBA NPs, the amount of free insulin in supernatants was assayed using UV–vis spectrophotometry. The drug loading efficiency was calculated using the equations below. Insulin entrapment efficiency (EE) and loading capacity (LC) were calculated using the following equations:

3 $${\text{Insulin}}\;{\text{loading}}\;{\text{capacity}}\,\left( {{\text{LC}}\% } \right) = \frac{{{{Total}}\;{{Insulin}} - {{Free}}\;{{Insulin}}}}{{{{Nanoparticles }}\;{{weight}}}} \times 100$$

4 $${{Insulin }}\;{{entrapment}}\;{{efficiency}}\,\left( {{{EE}}\% } \right) = \frac{{{{Total}}\;{{Insulin}} - {{Free}}\;{{Insulin}}}}{{{{Total}}\;{{Insulin}}}} \times 100$$

### In vitro drug release experiment

In vitro insulin release experiments were carried out. In order to simulate the in vivo conditions the temperature of 400 ml PBS in the oil bath was set at 36.5 °C. 40 ml of the refrigerator stored POSS-APBA@insulin solution is put in a dialysis tube of MWCO 8 k D and stabilized at room temperature. The charged dialysis tube was put in the PBS solution simulating in vivo condition. As a control, six 1.5 ml aliquots were taken every 10 min. for 1 h. without adding glucose to the PBS solution and evaluated for insulin release. The PBS solutions of different glucose concentrations, 1.0 mg/ml, 1.5 mg/ml and 2.0 mg/ml was achieved by adding 400 mg, 600 mg and 800 mg glucose to the blank PBS solution and the insulin release at the different glucose concentrations was evaluated from 1.5 ml aliquots taken every 10 min. for 1 h. The amount and concentration of the glucose in the PBS solutions were kept constant by replenishing with the same amount of the respective glucose containing PBS solutions. The insulin release was evaluated from the absorbance at 275 nm of the UV–Vis spectra taken in the 200–700 nm range.

### Reagents and antibodies

Anti-α-tubulin was purchased from Santa Cruz Biotechnology (Santa Cruz, CA), anti-Caspase 3 and anti-Caspase 9 were purchased from Cell Signaling Technology (Danvers, MA). HRP-tagged anti-rabbit and anti-mouse were purchased from Enzo Life Science (Farmingdale, NY). MTT reagent was purchased from Sigma Aldrich (St. Louis, MO).

### Cell Lines and culture condition

The HeLa cell and human dermal fibroblast (HDF) cell were purchased from the American Type Culture Collection (ATCC) and maintained in DMEM supplemented with 10% fetal bovine serum (FBS) and 1% penicillin/streptomycin. Human umbilical vein endothelial (HUVE) cell was kindly provided from Professor Chung-Hyun Cho (Seoul National University College of Medicine, Korea) and maintained in M199 supplemented with 10% fetal bovine serum (FBS) and 1% penicillin/streptomycin. These cells were maintained in humidified atmosphere containing 5% CO_2_ at 37 °C.

### Cell viability assay

Cells were seeded in 96-well culture plate and incubated in culture medium until 80% confluence. The cells were treated with various concentrations of samples for 24 h, and incubated with MTT reagent for 2 h. Blue formazan crystals were solubilized in DMSO, and formazan levels were determined at 570 nm using an Infinite M200 PRO plate reader (Tecan Group Ltd., Männedorf, Switzerland).

### Immunoblotting

Cells were washed with cold PBS, and then lysed in the triton lysis buffer containing protease and phosphatase inhibitors. After incubation for 30 min on ice, lysates were centrifuged at 13,000 rpm for 10 min at 4 °C, and supernatants were collected. Lysates were separated on 8–15% SDS–polyacrylamide gels, and transferred to nitrocellulose membranes (GE Healthcare Life Sciences, Chicago, Illinois). Membranes were blocked in 5% skim-milk for 1 h and incubated with primary antibodies overnight at 4 °C. Membranes were incubated with a horseradish peroxidase (HRP)-conjugated secondary antibodies for 1 h, and then visually evaluated by using the ECL detection kit (Biomax Co., Ltd, Seoul, Korea)^27^.

## Results and discussion

**Figure 1:**
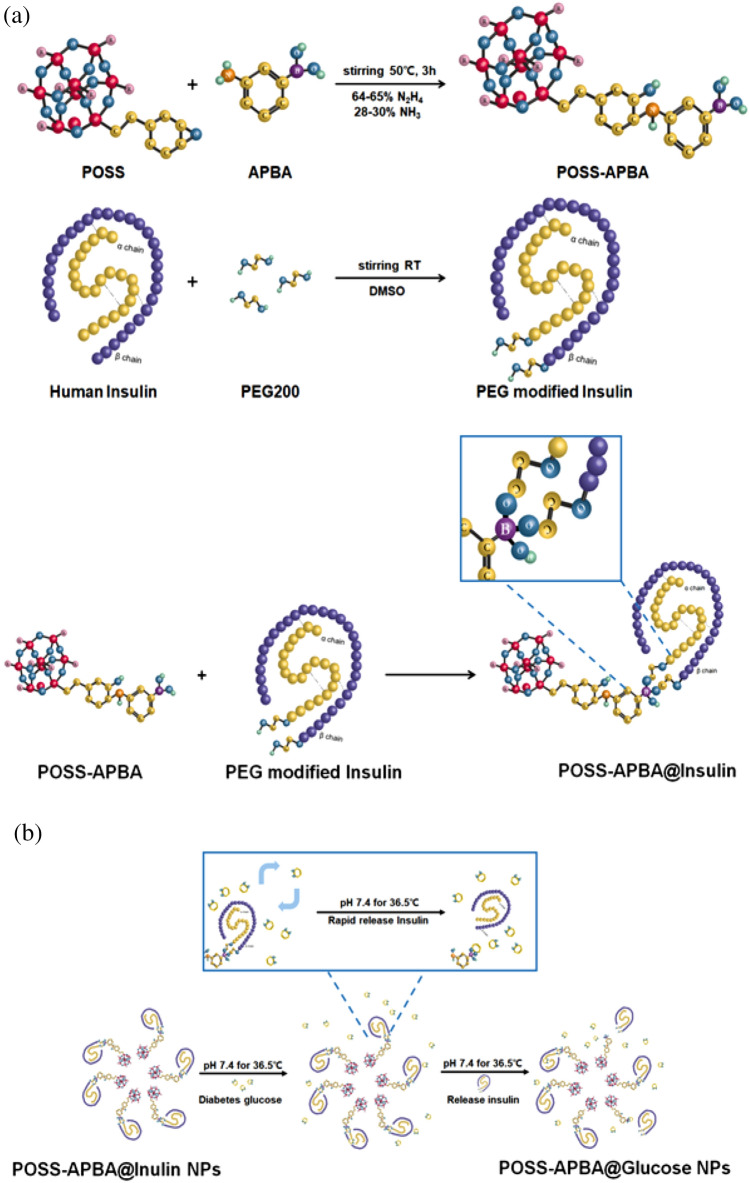
(**a**) Synthesis design of POSS-APBA, PEG modified insulin and POSS-APBA@insulin (**b**) Insulin releasing mechanism of glucose specific response by POSS-APBA@insulin at pH 7.4 at 36.5 °C.

POSS-APBA was synthesized from APBA and POSS to form -C-NH-bond using the epoxide group of POSS and the primary amine groups of the APBA. The diol-modified insulin self-assembled into core–shell structured micelles as a result of aggregation of the hydrophobic inner core with the hydrophilic hydroxyl groups of PEG forming the outer shell. The synthesis of POSS-APBA@insulin is shown in Fig. 1a. The synthesized POSS-APBA@insulin NPs bond with glucose under in vivo conditions and release insulin as shown in Fig. 1b. To confirm the interchange reaction, 10 mg/ml glucose solutions were added to POSS-APBA@insulin NPs and analyzed to confirm the release of insulin and formation of POSS-APBA@glucose.

**Figure 2:**
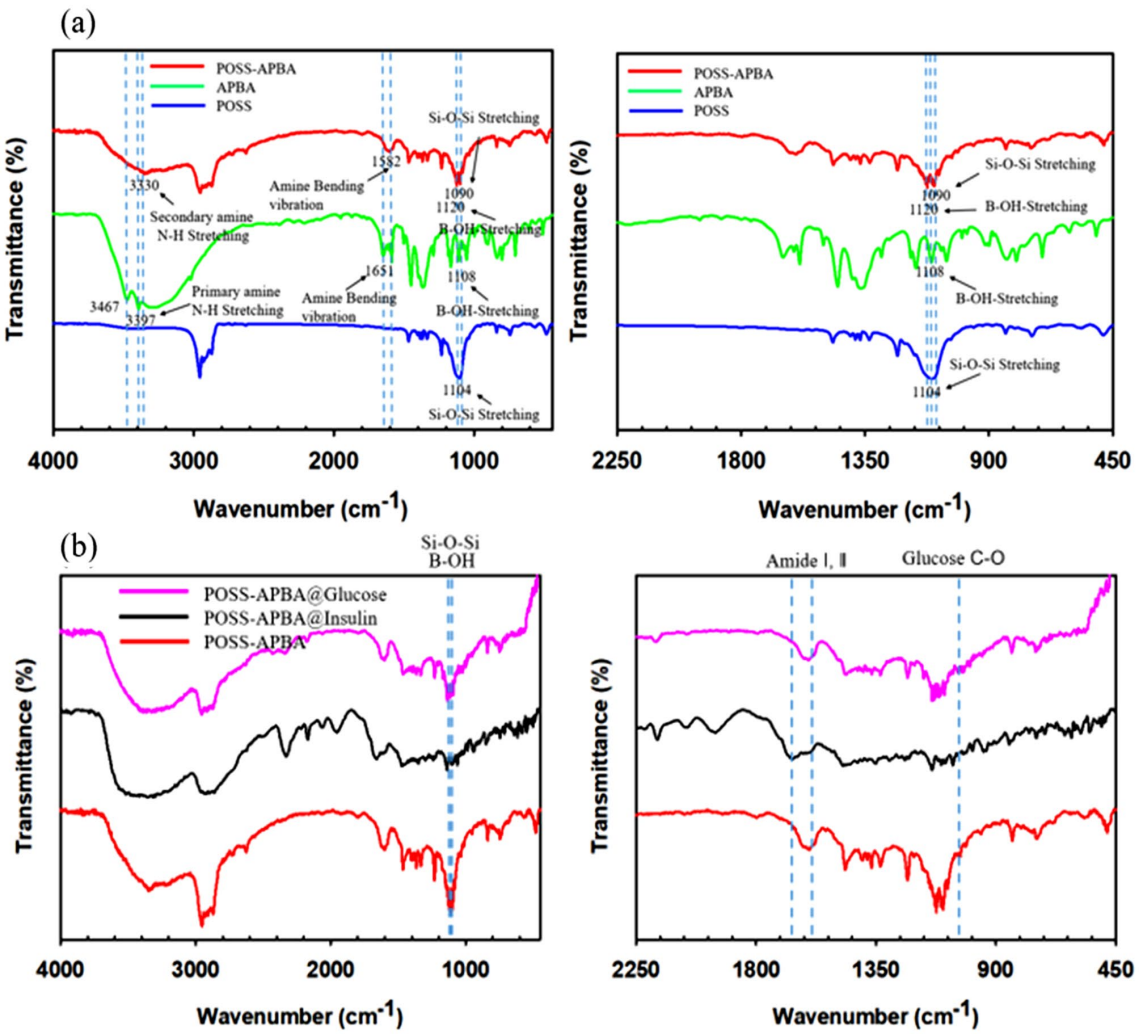
FT-IR spectra of (**a**) POSS, APBA, POSS-APBA (**b**) POSS-APBA@insulin modified NPs, POSS-APBA@insulin modified NPs after reaction with 10 mg/ml glucose.

The structure of the final product was confirmed by ^1^H NMR analysis (Supporting information Fig. S1) and FT-IR spectra (Fig. 2). In Fig. 2a, the peaks in the FT-IR spectra corresponding to POSS of the Si–O–Si stretching indicated at 1000–1300 cm^−1^. The C–N stretch of APBA and the POSS peak at 1240 cm^−1^ were seen due to the presence of C–O stretching vibrations of the epoxide group. The new peak at 1470 cm^−1^ in the POSS–APBA spectrum have confirmed caused by the C–N, C–N–C stretching, C–C stretching (phenyl ring), and B–O stretching of APBA, but these peaks are absent in the spectra of POSS. The band at 3350 cm^−1^ can be assigned to the N–H stretching of the secondary amine in POSS-APBA.

It also shows a broad peak at 3300 cm^−1^, the O–H stretching of POSS-APBA due to epoxide ring opening. In Fig. 2b, the peak of POSS-APBA@insulin completely appeared the amide I(~ 1660 cm^−1^), II (~ 1546 cm^−1^) absorption bands with contribution the α-helical protein due to the presence of insulin^28,29^. The release of insulin on addition of glucose can be confirmed by the disappearance of the Amide I, II peak (at about 1660, 1544 cm^−1^) of insulin. On bonding with glucose the spectra is similar to that of POSS-APBA, but the strong C-O vibration peak of glucose at 1035 cm^−1^ confirms the bonding of glucose to form POSS-APBA-Glucose.

**Figure 3:**
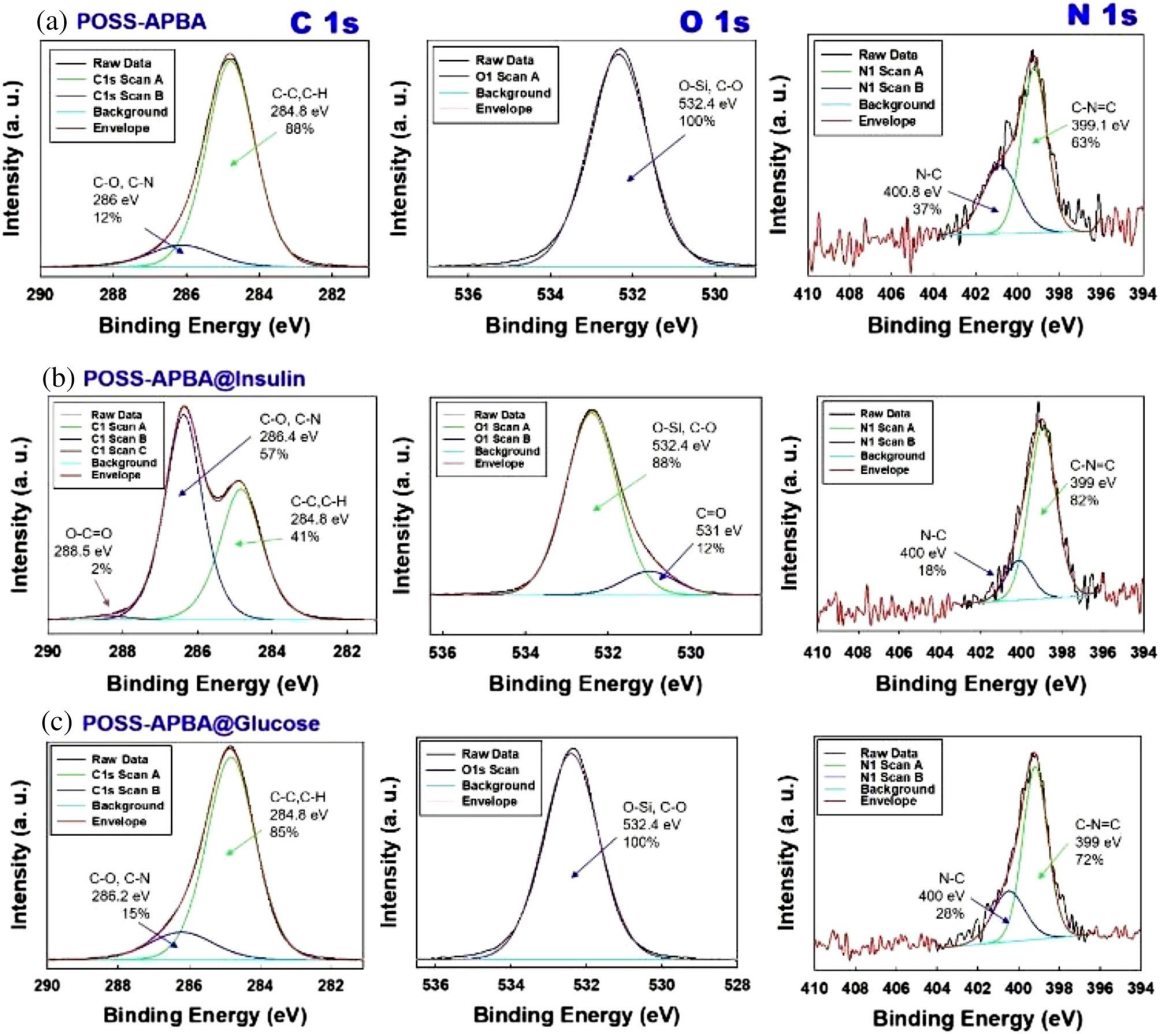
Deconvolution of high-resolution XPS spectra C1s, O1s and N1s peaks of (**a**) POSS-APBA NPs (**b**) POSS-APBA@insulin NPs and (**c**) POSS-APBA@glucose NPs.

Figure 3 shows the C1s, O1s, N1s peaks from XPS spectrum analysis of POSS-APBA NPs, POSS-APBA@insulin NPs, POSS-APBA@glucose NPs exhibiting the elemental composition, chemical state and the chemical bonding information. Only the characteristic C–C, C–H peaks (284.88 eV), C–O, C–N peaks (286.08 eV), O–Si, C–O peaks (532.28 eV), the N–C peak (400.78 eV), and the C–N=C (399.18 eV) of POSS-APBA are present suggesting that the POSS-APBA synthesized is of high purity without impurities. POSS-APBA@insulin NPs exhibits the amide C=O peaks of insulin protein with the C1s occurring at 288.48 eV (2%) and the O1s occurring at 530.98 eV (12%) on bonding with POSS-APBA. The presence of insulin can also be confirmed by the ratio of N–C and C–N=C in the N1_S_ peak. The characteristic peaks of insulin disappear on reaction of POSS-APBA@insulin NPs with glucose to form POSS-APBA@glucose NPs, and the O1s peak has the same bonding energy as POSS-APBA NPs but with a change in the peak ratio of C1_S_ and N1_S_ due to the bonding of the glucose with the diol.

**Figure 4:**
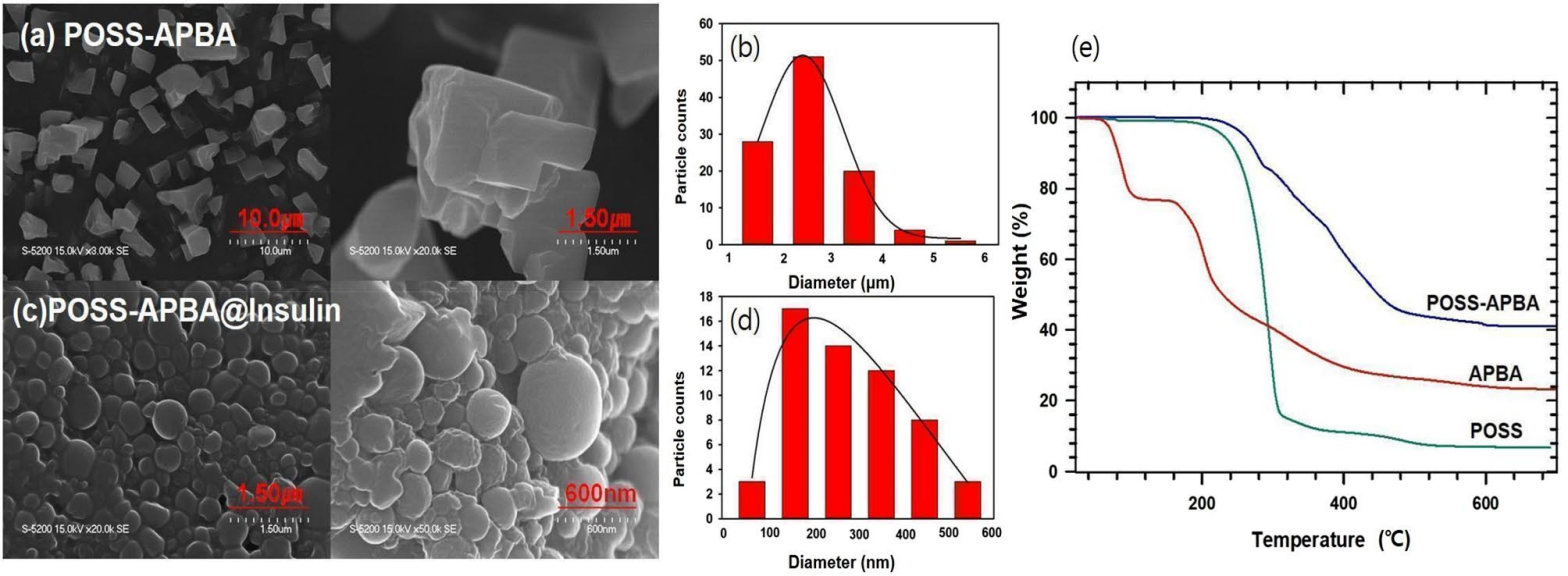
SEM images of (**a**) the POSS-APBA NPs and (**c**) POSS-APBA@Insulin NPs, the particle size distributions of (**b**) POSS-APBA NPs and (**d**) POSS-APBA@Insulin NPs, and TGA spectra (**e**) of POSS (green line), APBA (red line), POSS-APBA (blue line).

The POSS-APBA NPs exhibit a normal size distribution with the average value of 2.54 ± 0.8 μm with an octahedral structure, as can be seen in Fig. 4a and b. This is in concurrence with reports that irregular octahedral structure is formed due to the weak hydrogen bonding between OH and NH bonds^30,31^. Figure 4c and d shows the POSS-APBA@insulin NPs where the hydrogen bonding in POSS-APBA is broken and the bond between PEG diol on the insulin surface and boronic acid is formed resulting in self assembly to form spherical micelles. The average size of POSS-APBA@insulin NPs is 298 ± 121 nm with a normal size distribution as can be seen in Fig. 4d. It appears that the hydrophobic part of insulin bonds strongly with POSS and nano particles of defined size distribution is formed. It was confirmed by TEM (Supporting information Fig. S2). That the average particle size of POSS-APBA@glucose was approximately 50 nm from which insulin was released. This result could morphologically prove that insulin, a relatively large protein drug, causes an exchange reaction with glucose. To study the stability of the POSS-APBA@Insulin NPs, the size and size distribution of the freshly prepared NPs and NPs stored in 4 °C for more than 3 months were also observed at S3. The size and size variation of POSS-APBA@Insulin NPs for more than 3 months storage (233 ± 34 nm) was lower than initial during stored for a prolonged period of time. Thermogravimetric analysis (TGA) was applied to evaluate the thermal stability of pure POSS, APBA, and POSS-APBA. Para-substituted APBA isomers have poor heat stability at Fig. 4e. This causes decomposition to begin below 200 °C, with a mass loss of 50% at 225 °C. In addition, it is reported that boric acid tends to form B_2_O_3_ as it decomposes by heat, and this characteristic is known to affect the decomposition and flame retardant properties^32^. POSS-APBA remains stable up to 280 °C, and was decomposed very slowly as the temperature rises, leaving the half residual mass at 455 °C.

This suggests that it has higher thermal stability compared with POSS; that is, thermal decomposition occurs over a broader temperature range, suggesting the suppression of thermal decomposition on incorporation of POSS macromers^33^. POSS-APBA displayed increased residues of about 18% (i.e., char and ceramic yields).

The insulin entrapment efficiency (EE) of the prepared POSS-APBA@insulin is 73.2% due to the high hydrophobic nature of POSS resulting in strong bonding with insulin. The loading capacity of POSS-APBA@insulin is 50.5%.

**Figure 5:**
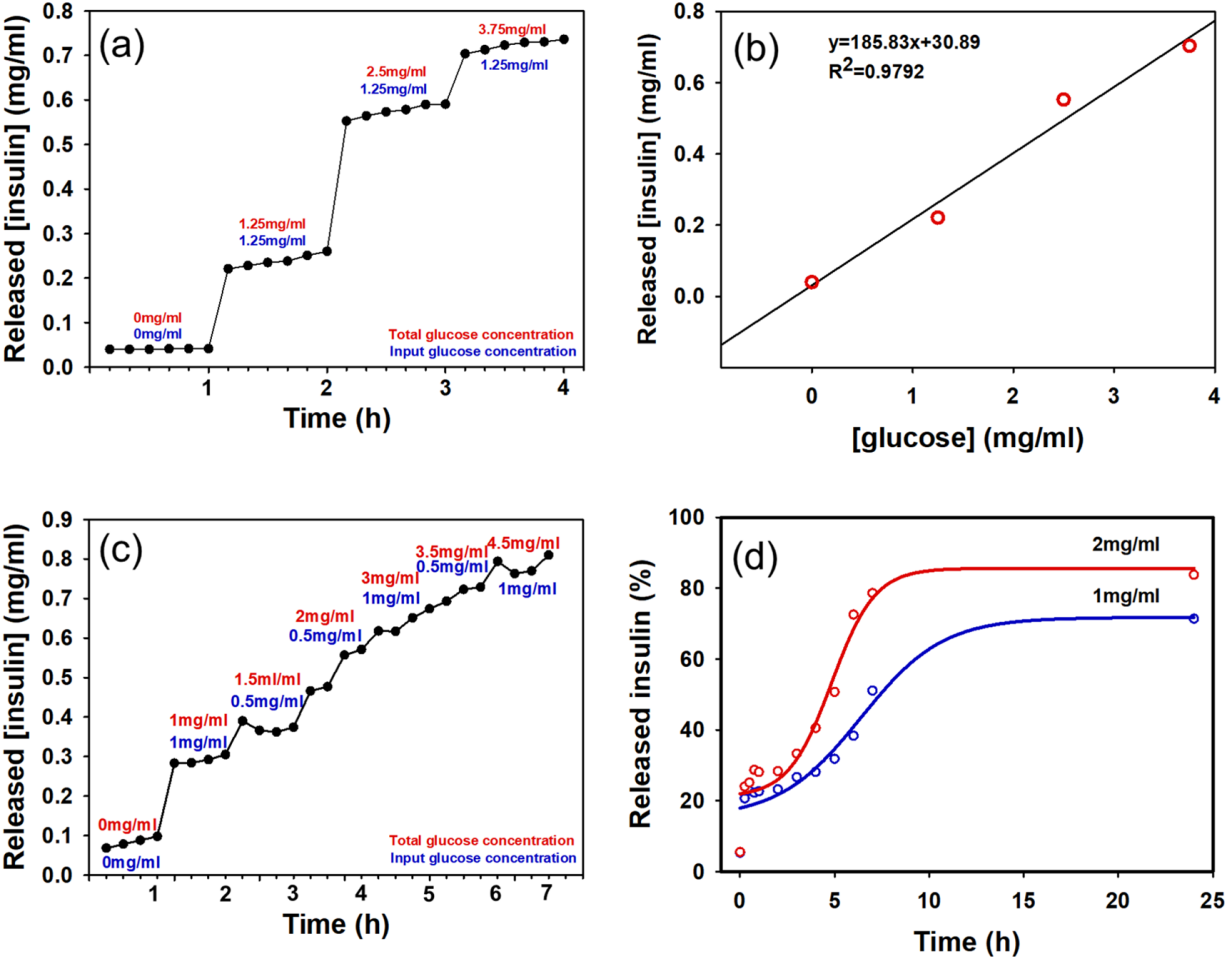
In vitro glucose-responsive release of insulin from POSS-APBA@Insulin NPs. (**a**) and (**c**) continual insulin release, (**b**) typical insulin release curves at glucose concentrations of 0–3.75 mg/mL and (**d**) insulin release at glucose concentration of 1 mg/mL and 2 mg/mL (pH 7.4) at 37 °C.

To study the insulin release kinetics of POSS-APBA@insulin NPs, they were incubated in a PBS buffer of pH 7.4 at 37 °C. In Fig. 5d, the glucose concentration of 1 mg/mL (1 g/L, 100 mg/dL) is the normal glucose concentration level and the typical diabetic glucose level of hypoglycemia patients can be 2 mg/mL (2 g/L, 200 mg/dL) or over. Up to present, high glucose concentrations of 400 mg/ml is required for insulin release^34^ and the reaction sensitivity is low in that insulin is released only after 2 h. We confirmed that the insulin released about 60% and 80% at a glucose concentrations of 1 mg/mL and 2 mg/mL at 24 h. The rest of insulin have remained POSS-APBA@insulin form without coming out of the dialysis tube. From the insulin release data in Fig. 5a and c where the concentration of glucose is increased hourly, we can see that insulin is released even at a low glucose concentration of 1 mg/ml. Insulin is not released from POSS-APBA@insulin in the absence of glucose. The insulin is released within 10 min. after addition of glucose and equilibrates in 30 min, exhibiting quick response.

It appears that the bulky nature of POSS provides easier access of the glucose to the boronic acid groups^35^. The insulin release of POSS-APBA@insulin NPs is shown Fig. 5b. The release is linearly proportional to the glucose concentration, y = 185.83x + 30.89 and R^2^ = 0.9792. These results suggest that POSS-APBA@insulin NPs may very probably applicable as a smart drug delivery system.

**Figure 6:**
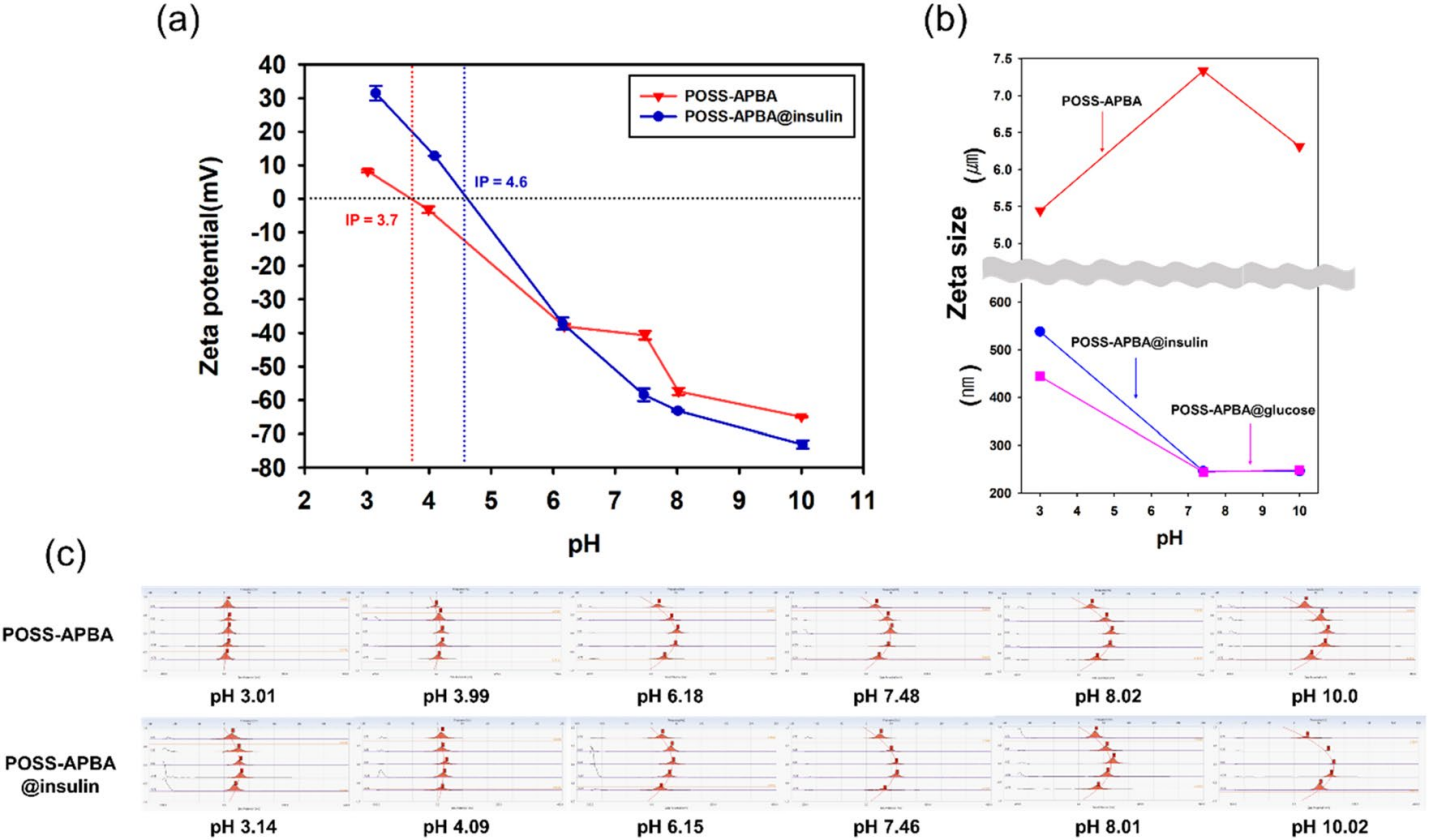
Variation of zeta potential (mV) (**a**), Zeta size and (**b**) Electro-Osmosis plot (**c**) with the change in pH values for POSS-APBA, POSS-APBA@-Insulin NPs, POSS-APBA@glucose NPs (only zeta size).

Table 1 and Fig. 6a shows that the zeta potential decreases from 8.23 to − 64.97 mV when pH is increased from 3.1 to 10.0. Proton is released from the APBA surface which is positively charged at pH 3.7 as the pH is increased above the isoelectric point (Ip) and the phenyl boronic acid group of APBA becomes negatively charged. In the neutral pH state the zeta potential becomes − 40.69 mV, existing as a highly stable colloidal dispersions. The POSS-APBA@insulin NPs also exhibits a similar behavior. The zeta potential of POSS-APBA@insulin NPs decreases from 31.44 to − 73.14 mV as the pH is raised from 3.1 to 10.0. The change is higher than POSS-APBA suggesting higher stability of the colloid at 37 °C. The Ip of POSS-APBA@insulin NPs is pH 4.6 and the zeta potential at the neutral pH is -58.42 mV. PBA is generally known to exist as a trigonal –B(OH)_2_ structure at pKa below 8.6 and a tetrahedral –B(OH)_3_ structure above pKa 8.6 and also known to form stable complexes with polyols^36^. PBA itself can not be used as a drug release agent in a physiologic environment.

**Table 1:** Zeta potential (mV), zeta size (nm), PDI, conductivity (mS/cm), mobility (cm^2^/Vs) of POSS-APBA and POSS-APBA@insulin at 37 °C.

Sample	pH	Zeta potential (mV)	Mobility (cm^2^/Vs)	Conductivity (mS/cm)
POSS-APBA	30.1	8.235	6.42E-05	0.9774
	3.99	− 3.275	− 2.6E-05	0.52755
	6.18	− 38.045	− 3E-04	0.124
	7.48	− 40.695	− 3E-04	0.1375
	8.02	− 57.435	− 4E-04	0.20685
	10	− 64.97	− 5E-04	0.282
POSS-APBA-insulin	3.14	31.44	2E-04	0.37235
	4.09	31.44	1E-04	0.07615
	6.15	− 37.17	− 3E-04	0.0639
	7.46	− 58.425	− 5E-04	0.1344
	8.01	− 63.195	− 5E-04	0.1902
	10.02	− 73.14	− 6E-04	0.32375

This has been overcome by employing the POSS-APBA and POSS-APBA@insulin NPs prepared in this study, which exist as a highly stable negatively charged dispersion in the neutral pH. Thus it appears that the stable negative charge of the boronic acid can selectively bond with glucose to allow fast reaction. Figure 6b shows the change in zeta size at pH 3.0, 7.5, 10.0 of POSS-APBA NPs, POSS-APBA@insulin NPs, and POSS-APBA@glucose NPs. As was seen in the SEM data of Fig. 4a and c, POSS-APBA becomes nano particles as insulin is loaded to form POSS-APBA@insulin. It appears that the hydrophobic insulin introduces orientation to the amphipathic structure of telechelic POSS-APBA to result in a stable colloidal behavior. The particle size of POSS-APBA is large at neutral pH, but the particle size of POSS-APBA@insulin decreases as pH is increased. Similar behavior is seen in the case of POSS-APBA@glucose. This appears to be due to the stabilization through bonding of the boronic acid with glucose or PEG modified insulin, to result in high stability (over ± 30 mV) of the dispersion at pHs 3, 7, 10^26^. Table 1 shows the change in the zeta potential, zeta size, PDI, mobility, and conductivity of POSS-APBA and POSS-APBA@insulin with pH. Figure 6c shows the change in the electro-osmosis plot of POSS-APBA and POSS-APBA@insulin with pH. Electro-osmosis is the liquid flow induced by the applied potential across the cell in zeta potential measurement. The mobility of POSS-APBA increases with increase in pH over 10 times and the mobility of POSS-APBA@insulin increase about 2 times.

**Figure 7:**
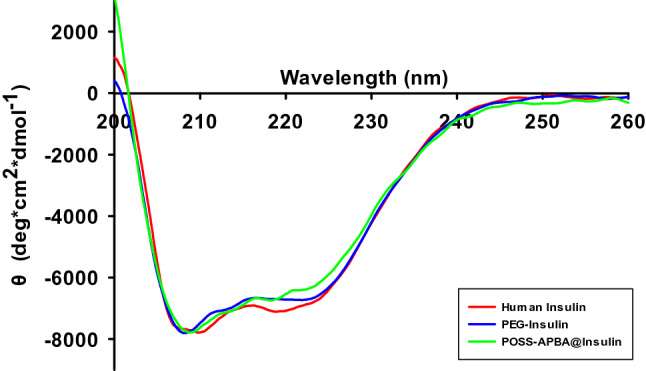
Circular dichroism (CD) spectra of human insulin (red line), PEG-insulin (blue line), POSS-APBA@insulin (green line).

The DMSO-solvated insulin was an ensemble of conformations with a residual α-helical structure (making them as compact as they can probably be) and polyproline II helix. Effects of DMSO-specific solvation and conformation-restricting covalent structure was noted to play important roles in stabilizing the disordered state of the protein^37^. The successful preparation of POSS-APBA@insulin nanoparticles has already been validated in previous analysis. However, as it is of utmost importance to maintain the original structure of insulin during the fabrication of nanoparticles such that the activity of insulin is not decreased, we studied the secondary structure of PEG-insulin and POSS-APBA@insulin nanoparticles using circular dichroism spectroscopy (CD) to establish the presence of the α-helix, and β-sheets secondary structure, characteristic of insuline, in PEG-insulin and POSS-APBA@insulin nanoparticles, and thus validate that the secondary structure of insulin has not been denatured and that the activity is maintained^38,39^. Fig. 7 shows the CD spectra of insulin, PEG-insulin, and POSS-APBA@insulin. The red line analyzing the secondary structure of insulin used in this study displays the α-helix, β-sheets peaks^40^. This confirms the activity of insulin used in this study and thus it can be used as a control sample. The blue line and green line in Fig. 7 both show the characteristic peaks of insulin secondary structure and the overall shape of the lines are very similar to the red line of insulin. This suggests that the secondary structure of insulin is not denatured during the preparation of PEG-insulin and POSS-APBA@insulin and that the activity of insulin is maintained even after the preparation of the nanoparticles. Analysis was also carried out after applying a deconvolution program, Spectra Manager version 2. Table 2 shows the % composition of α-helix, β-sheets, β-turn, and random coil in the secondary structure of insulin. The compositions of α-helix, β-sheets, β-turn, and random coil were all very similar. This can be an evidence that the secondary structure of insulin is not denatured by the incorporation of PEG or POSS-APBA. Ultimately, this shows that the secondary structure of insulin is not affected in the preparation of PEG-insulin or POSS-APBA@insulin and that the activity of insulin is maintained.

**Table 2:** Secondary structure elements of human insulin, PEG-insulin, POSS-APBA@insulin calculated by CD analysis.

	Human Insulin	PEG-Insulin	POSS-APBA@Insulin
% Helix	29.1	27.1	27.6
% Beta	16.5	16.5	23.7
% Turn	23.8	23.3	20.5
% Random	30.5	33.1	28.1
% Total	100	100	100

Expressed the percent of total secondary structure elements.

**Figure 8:**
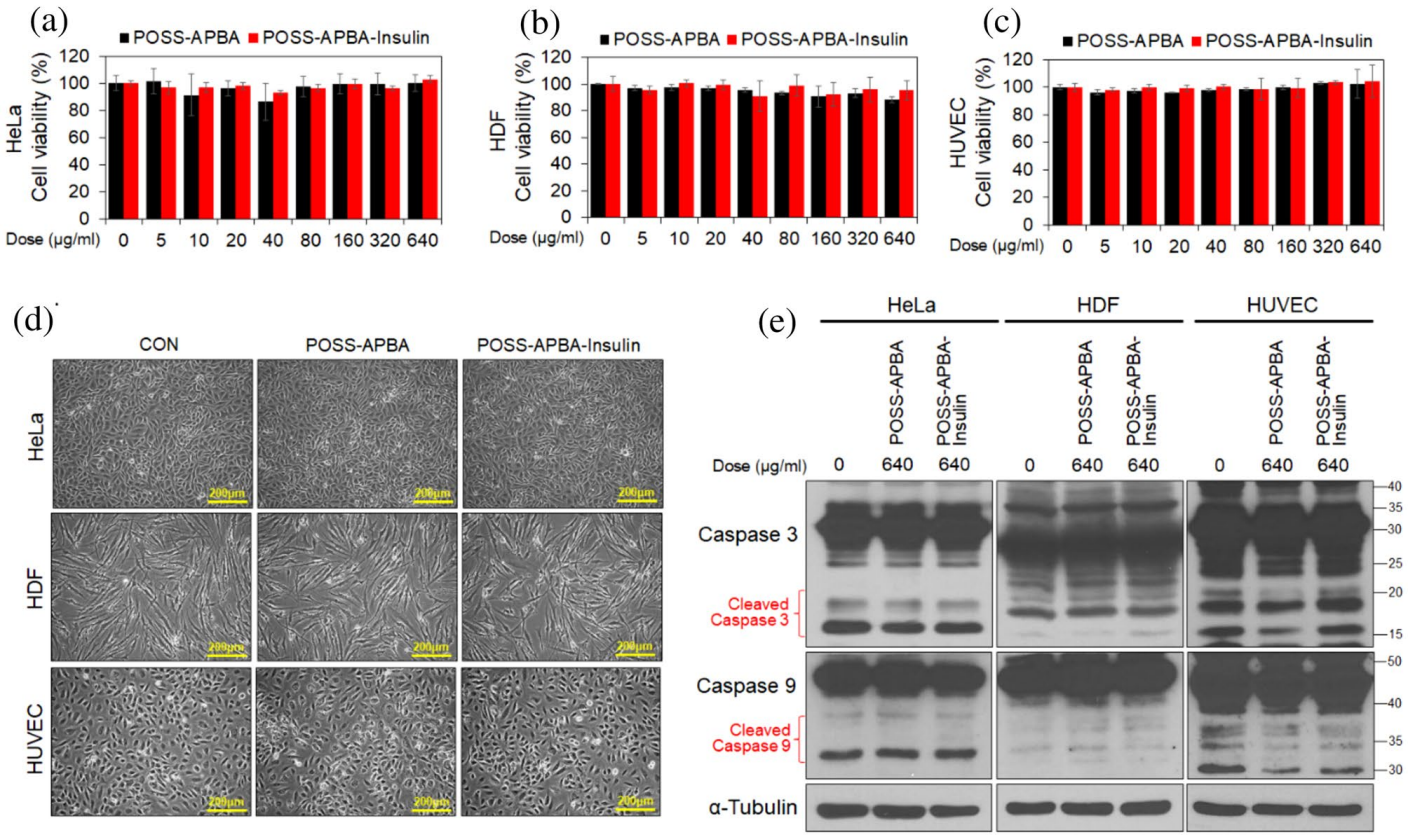
HeLa, HDF and HUVE cells treated with various concentrations of POSS-APBA and POSS-APBA@insulin for 24 h, (**a–c**) respectively. Cell viability was measured by MTT assay. Micrographs of cell morphology (**d**) of HeLa, HDF and HUVE cells treated with 640 μg/ml of POSS-APBA and POSS-APBA@insulin for 24 h. Immunoblotting results of cell lysates (**e**). Full-length gels and blots are included in the Supporting Information 4.

To apply our drug delivery system to cells, we performed cytotoxicity experiments on POSS-APBA and POSS-APBA@insulin NPs using HeLa, HDF and HUVE cells. It was confirmed that both POSS-APBA and POSS-APBA@insulin NPs are not cytotoxic up to 640 μg/ml concentration, Fig. 8a–c, and that there is no morphological change of cells at 640 μg/ml concentration, Fig. 8d. Furthermore, when confirming intracellular apoptosis signaling, cleaved caspase 3 and caspase 9 are not increased by POSS-APBA and POSS-APBA@insulin at 640 μg/ml concentration in HeLa, HDF and HUVE cells, Fig. 8e. These results suggest that POSS-APBA and POSS-APBA@insulin NPs have the potential to be applied to cells.

## Conclusion

The phenylboronic acid-functionalized POSS and POSS-APBA@insulin have been successfully fabricated as glucose-responsive system. The amphiphilic oligomer allowed self-assembly of POSS-APBA@insulin into micelles of spherical shape with core–shell nano structure, and are well-dispersed as shown by Zeta-potential analysis and uniform in size distribution exhibiting 298 ± 121 nm size range in SEM and TEM. Utilizing POSS-APBA@insulin, vesicles can be formed with a high insulin entrapment efficiency of 73.2% and loading capacity of 50.5%. POSS-APBA@insulin exhibits the obvious glucose-responsive behaviors and the response is proportional to glucose concentration, y = 185.83X + 30.89, R square value = 0.9792. Glucose binding increases the total fraction of ionized boronic acid, particularly at neutral pH. Furthermore, when confirming intracellular apoptosis signaling, cleaved caspase 3 and caspase 9 are not increased by up to 640 μg/ml POSS-APBA or POSS-APBA@insulin in HeLa, HDF and HUVE cells. Application in the biomedical field for controlled delivery of insulin appears to be promising.

## Supplementary Information

Supplementary Information.
