# Author Correction: Insulin smart drug delivery nanoparticles of aminophenylboronic acid–POSS molecule at neutral pH

**DOI:** 10.1038/s41598-021-03340-6

**Published:** 2021-12-07

**Authors:** Won Jung Kim, Yong-Jin Kwon, Chung-Hyun Cho, Sang-Kyu Ye, Kyu Oh Kim

**Affiliations:** 1 Department of Fiber-System Engineering, Dankook University, 152, Jookjeon-ro, Suji-gu, Yongin-si, Gyeonggi-do 448-701 Republic of Korea; 2 Departments of Pharmacology, Seoul National University College of Medicine, Seoul, South Korea

Correction to: *Scientific Reports* 10.1038/s41598-021-01216-3, published online 08 November 2021.

The original version of this Article contained errors.

The spelling of the author Yong-Jin Kwon was incorrectly given as Yong Jin Kwon.

In the Materials and Methods section, under the subheading ‘Synthesis of PEG-insulin’,

“To introduce diol groups to insulin, 8.0 mg insulin and 1.0 mg PEG were dissolved in 10 ml DMSO to which 0.1–0.2% sodium cyanoborohydride catalyst was added and stirred for 1 h. then stabilized at room temperature for 1 h.”

now reads:

“To introduce diol groups to insulin, 8.0 mg insulin and 1.0 g PEG were dissolved in 10 ml DMSO to which 0.1–0.2% sodium cyanoborohydride catalyst was added and stirred for 1 h. then stabilized at room temperature for 1 h.”

The original Article and accompanying Supplementary Information file have been corrected.

