# Supplementary material for: Insulin smart drug delivery nanoparticles of aminophenylboronic acid–POSS molecule at neutral pH
Source: Sci Rep. 2021 Nov 8;11:21894. doi: 10.1038/s41598-021-01216-3 (PMC8575987; doi:10.1038/s41598-021-01216-3)
Supplement: Supplementary file 1 — Supplementary Information. [file 41598_2021_1216_MOESM1_ESM.pdf]

Supporting Information

Insulin Smart Drug Delivery Nanoparticles of Aminophenylboronic acid–POSS Molecule at Neutral pH

Won jung Kim[1], Yong-Jin Kwon[2], Chung-Hyun Cho[2], Sang-Kyu Ye[2,], Kyu oh Kim[1,*]*

[1]Department of Fiber-system Engineering, Dankook University, 152, Jookjeon-ro, Suji-gu, Yongin-si, Gyeonggi-do, 448-701, Republic of Korea

[2]Departments of Pharmacology, Seoul National University College of Medicine,

*Corresponding author with contact details:

• E-mail address: affablekim@gmail.com (K.O. KIM) ; sangkyu@snu.ac.kr (S.K.Ye)

•Tel.: +82 31 8005 3561; Fax: +82 31 8005 3564

POSS-APBA

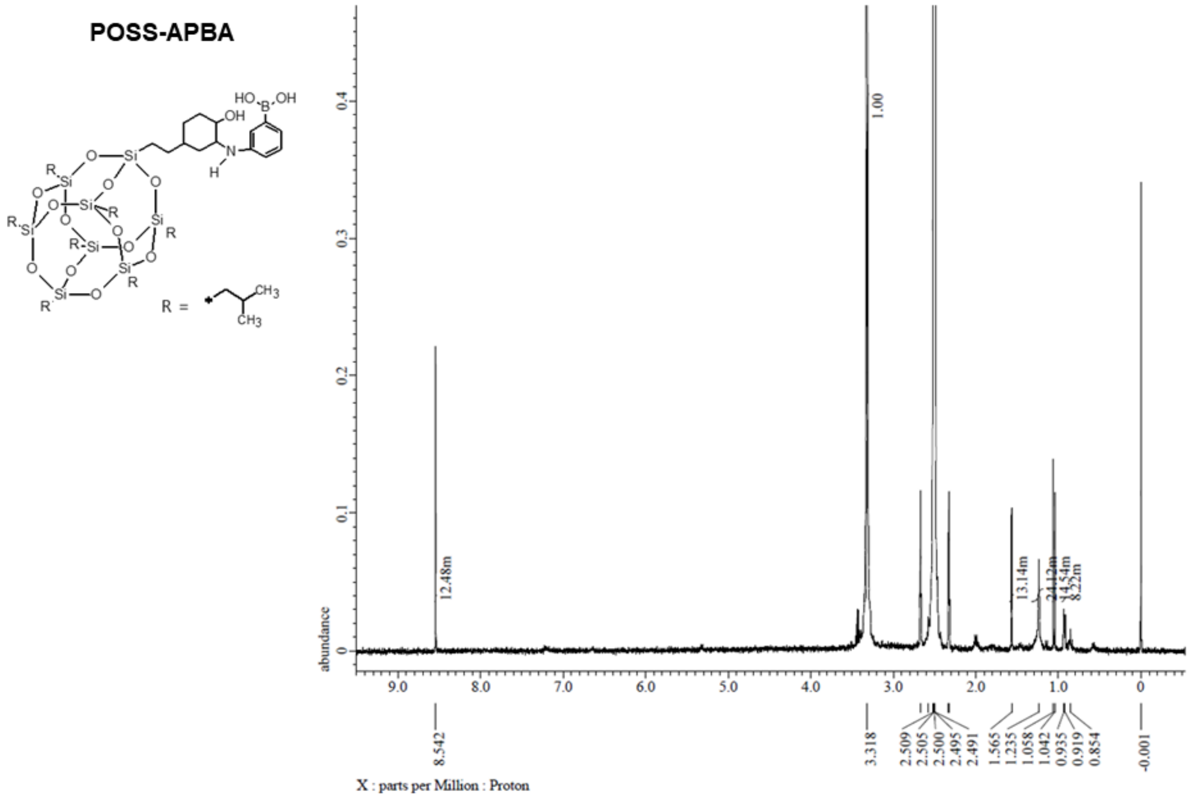

POSS-APBA@insulin

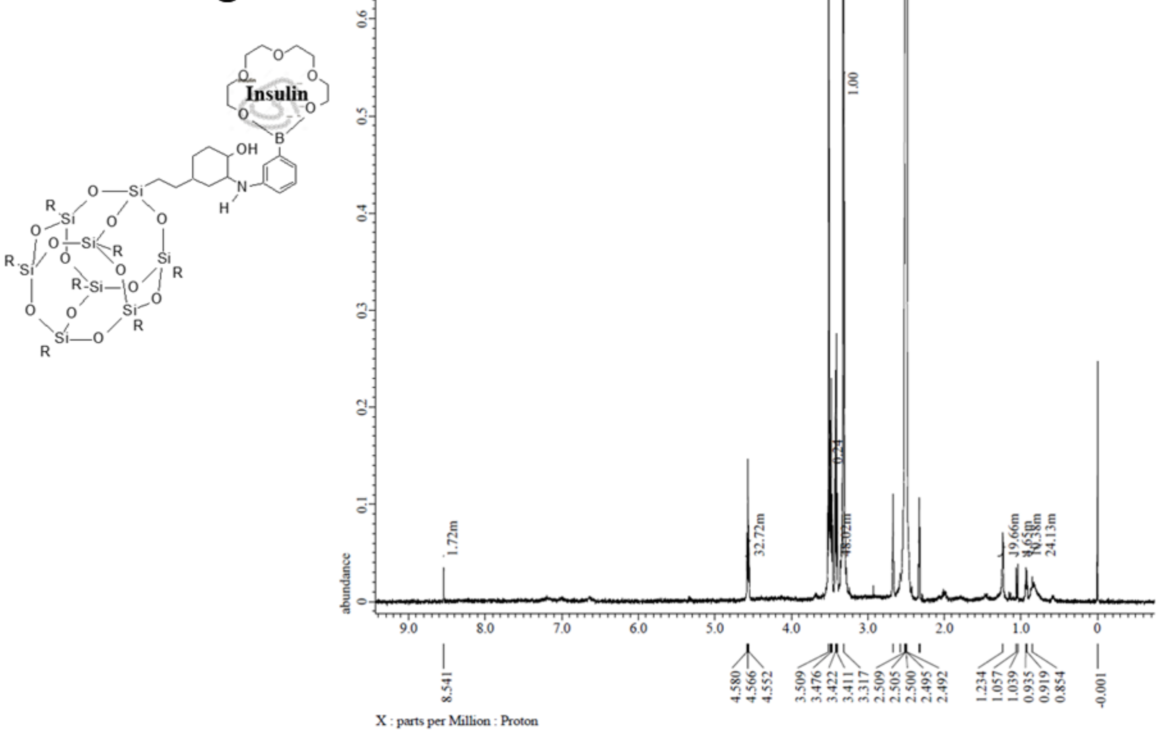

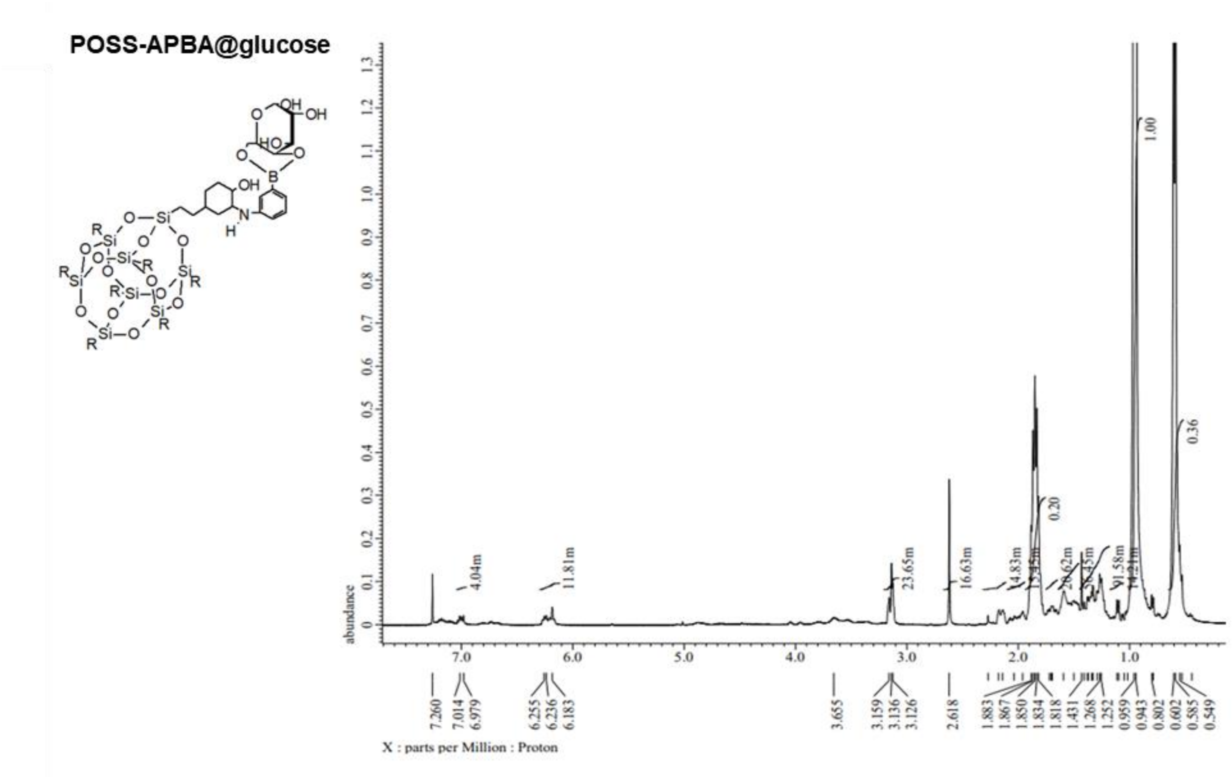

Supporting Information 1. ^{1}H-NMR spectra: POSS-APBA, POSS-APBA@insulin, POSS-APBA @glucose spectrum.

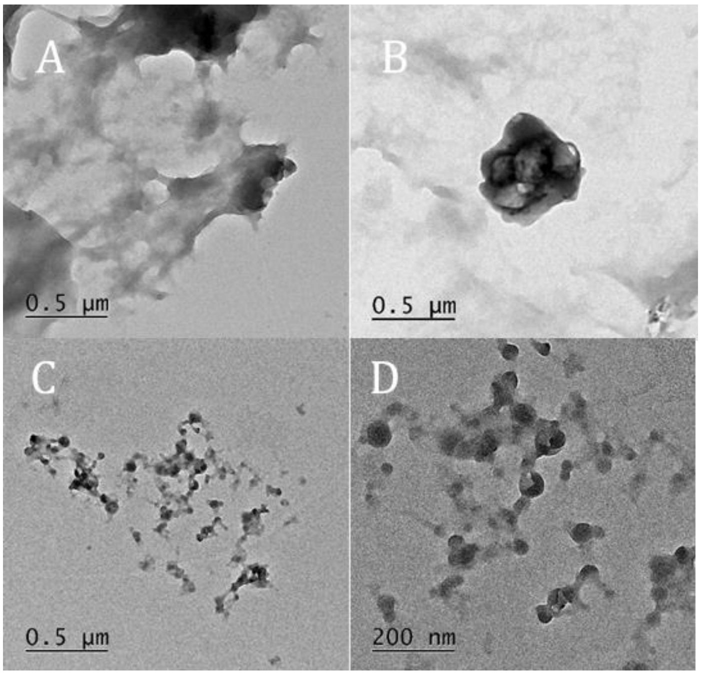

Supporting Information 2. TEM images of POSS-APBA@insulin (A)(B), POSS-APBA@glucose after releasing insulin (C)(D)

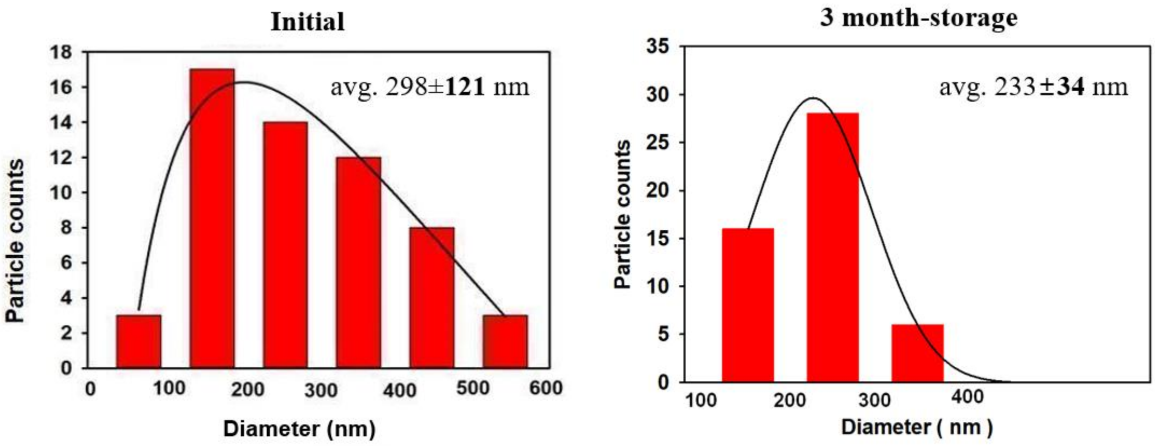

Supporting Information 3. Storage stability study

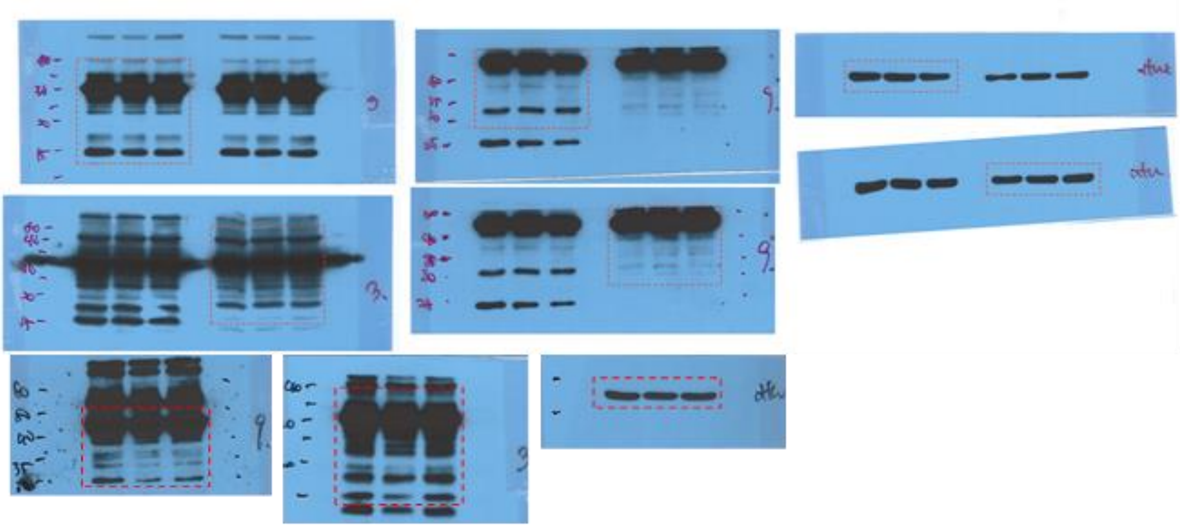

Supporting Information 4. Full-length blots in figure 8(e). The red dotted lines are the main figures.
